# Child–Robot Relationship Formation: A Narrative Review of Empirical Research

**DOI:** 10.1007/s12369-019-00569-0

**Published:** 2019-07-03

**Authors:** Caroline L. van Straten, Jochen Peter, Rinaldo Kühne

**Affiliations:** Amsterdam School of Communication Research (ASCoR), University of Amsterdam, P.O. Box 1579, 1001 NG Amsterdam, The Netherlands

**Keywords:** Child–robot interaction, Human–robot interaction, Artificial intelligence, Automation, New-ontological-category hypothesis

## Abstract

This narrative review aimed to elucidate which robot-related characteristics predict relationship formation between typically-developing children and social robots in terms of closeness and trust. Moreover, we wanted to know to what extent relationship formation can be explained by children’s experiential and cognitive states during interaction with a robot. We reviewed 86 journal articles and conference proceedings published between 2000 and 2017. In terms of predictors, robots’ responsiveness and role, as well as strategic and emotional interaction between robot and child, increased closeness between the child and the robot. Findings about whether robot features predict children’s trust in robots were inconsistent. In terms of children’s experiential and cognitive states during interaction with a robot, robot characteristics and interaction styles were associated with two experiential states: engagement and enjoyment/liking. The literature hardly addressed the impact of experiential and cognitive states on closeness and trust. Comparisons of children’s interactions with robots, adults, and objects showed that robots are perceived as neither animate nor inanimate, and that they are entities with whom children will likely form social relationships. Younger children experienced more enjoyment, were less sensitive to a robot’s interaction style, and were more prone to anthropomorphic tendencies and effects than older children. Tailoring a robot’s sex to that of a child mainly appealed to boys.

**Electronic supplementary material:**

The online version of this article (10.1007/s12369-019-00569-0) contains supplementary material, which is available to authorized users.

## Introduction

In recent years, robots have started to enter our homes and service environments. They are no longer used only for manufacturing, but also fulfill—as social robots—roles as assistants or companions [56, 141]. Social robots are typically user-friendly: Even people without technological training can use them. One group without technological training that is particularly likely to use social robots are children [116], who already encounter social robots in school, therapy, and entertainment settings. In this context, some scholars have pointed out beneficial effects of social robots on children’s learning [e.g., 135], social behavior [e.g., 64], and emotional wellbeing [e.g., 13]. Other scholars, in contrast, have voiced concerns about possibly detrimental developmental consequences of child–robot interaction (CRI) [25], notably, with respect to the crucial developmental task of forming meaningful relationships [14]. For example, researchers have pointed out that children may be deceived about the genuineness of the child–robot relationship, which may elicit emotional or psychological damage [126]. Moreover, children may establish master–servant relationships with robots, resulting in an immoral treatment of robots that may spill over to children’s treatment of peers [56].

Despite these contrasting views on the consequences of CRI, we lack a comprehensive overview of whether and how CRI affects the formation of child–robot relationships. Moreover, systematic knowledge about the determinants and underlying processes that play a role in the establishment of child–robot relationships is missing. Multiple CRI reviews have appeared, but they are rather limited in their scope and focus, dealing with specific social robot applications in educational settings [e.g., 125, 137], autism therapy [e.g., 12, 26], or physical therapy for disabled children [e.g., 144]. Other reviews have assessed CRI only in the broader context of human–robot interaction (HRI) [25, 41, 84, 151]. Finally, one study did survey some research on child–robot relationship formation, but does not present a comprehensive, systematic review of the field [i.e., 56].

Against this background, the purpose of the present review study is to gain insight into the predictors and mechanisms of relationship formation between children and social robots. We define social robots as robots that interact with us in a personal manner, enabling us to relate to them and empathize with them [22]. The emergence of child–robot relationships will be assessed through two concepts that are central to relationship formation: closeness and trust [16]. Closeness represents a feeling of connectedness or intimacy that could potentially result in the development of a friendship [131]. Trust comprises the expectancy that another person will keep his/her word or promises [113], as well as the reliance on another person’s knowledge and intent [74]. As previous studies tend to investigate the development of child–robot relationships often idiosyncratically, it seems worthwhile to organize the research by integrating individual findings into an overarching framework. We will also supplement our review with a methodological comparison of the studies, in order to provide crucial insights into the internal and external validity of findings.

## Overarching Framework

The present review organizes studies that are relevant to the topic of child–robot relationship formation along the lines of the Stimulus–Organism–Response (S–O–R) paradigm [51]. This paradigm posits that the influence of external events (i.e., stimuli), or more generally predictors, on outcome variables (i.e., responses) is mediated by internal states (i.e., of the organism) [91]. The paradigm forms the basis of existing models in HRI [i.e., 66, 77, 80]. These models all contain outcome variables that are relevant to relationship formation, such as trust and relationship satisfaction [66], as well as social attraction [77, 80]. Moreover, each of the existing HRI models incorporates robot characteristics as predictors, for example the robot’s personality [80], its physical versus virtual embodiment [77], or the robot’s role during the interaction [66]. Finally, all HRI models assume robot characteristics to elicit specific internal states, such as social presence, which are thought to mediate the influence of robot characteristics on the outcome variables.

The S–O–R paradigm thus seems to be a suitable heuristic to organize the literature in this review: ‘S’ represents the predictors of child–robot relationship formation, ‘O’ stands for potential mediators, and ‘R’ for the focal outcome variables, closeness and trust. Because our focus limits the relevant outcome variables to trust and closeness, we will center on the assessment of variables belonging to the ‘S’ and ‘O’ parts of the model and their role in explaining trust and closeness. As the field of HRI research is still evolving and methodologically heterogeneous [11], a quantitative meta-analysis seems currently premature [107]. Therefore, we abstain from reporting effect sizes and statistical analyses. The S–O–R model thus presents a conceptual tool to structure current knowledge about child–robot relationship formation rather than a statistical model to be tested.

Although not concerned with statistical testing, we do classify relevant variables in the literature into predictors, mediators, and outcomes. This quantitative terminology not only helps us to adequately represent the reviewed research, of which a large part is quantitative, but also aids us to structure the results of the narrative review transparently.

### Predictors of Child–Robot Relationship Formation

Previous research has focused on the examination of two dimensions in HRI: robot characteristics (i.e., robots’ character, physical capacities, and behavior) and interaction styles (i.e., the content of robots’ contributions to the interaction) [24]. Several of these characteristics and styles are omnipresent in studies both on HRI and on interpersonal relationships, and may also be expected to play a role in CRI. Therefore, they will guide our discussion of the predictors of child–robot relationship formation.

Dautenhahn [33] mentions four robot characteristics that are conducive to the formation of human–robot relationships. First, a companion robot should be tailorable to its user in terms of appearance and behavior. This is in line with the finding that children form relationships with individuals who are similar to themselves, both in terms of appearance and behavior [e.g., 16, 32, 43, 48, 100]. Positive effects of both types of similarity have also been found in CRI studies [27, 44].

Second, a robot’s functionality should be tailorable to match the user’s preferences, and allow for an expansion of the robot’s range of skills over time. This capacity has been confirmed to encourage human–robot social bonding [79]. Third, robots should adopt a humanlike rather than machinelike role and interact accordingly. Krämer et al. [72] acknowledge the importance of humanlike behavior, but argue that more specific roles should be determined based on the users’ perception.

Fourth and finally, companion robots need to possess social skills, which allow for socially interactive behavior and are essential to long-term robot acceptance. General [e.g., 16, 40, 45] as well as child-oriented [e.g., 32, 100] research on interpersonal relationship formation has pointed to the importance of two specific social skills: responsiveness and expressiveness. HRI studies have confirmed the relevance of these skills to the establishment of human–robot relationships [e.g., 19, 92]. Responsiveness refers to the probability that an interaction partner will (appropriately) respond to the other’s communicative behaviors [34]. Expressiveness consists of an emotional and a social dimension [110]: Emotional expressiveness refers to the ability to express emotions, attitudes, and interpersonal orientation cues spontaneously and accurately. Social expressiveness captures general speaking skills and the ability to engage interaction partners. Both responsiveness and expressiveness apply to verbal and nonverbal behavior [34, 110].

In addition to the abovementioned characteristics, the literature has devoted major attention to the influences that different types of robot embodiment have [e.g., 63, 87]. Therefore, we add embodiment as a final robot characteristic. Embodiment can be defined as referring to “the fact that a particular agent is realized as a physical robot or as a simulated agent” [108, p. 649]. In sum, we will focus on six robot characteristics: (a) responsiveness; (b) expressiveness; (c) tailoring of appearance and behavior; (d) tailoring of functionality; (e) role; and (f) embodiment.

As for the second crucial dimension in HRI research—interaction styles—it is conceivable that at least three types of interaction influence the process of relationship formation. First, relationships benefit from a development of routine interaction into strategic interaction, which aims at advancing a relationship (e.g., through self-disclosure of increasing depth and breadth) [16, 84]. Second, the expression of emotions is essential to the emergence of closeness and trust [15, 16, 146]. Therefore, emotional interaction is crucial for the development of a relationship. Third, the development of relationships requires time. Accordingly, memory-based interaction referring to shared knowledge and events in the past plays an important role in relationship formation [16], and, in our view, deserves to be treated as an interaction style of its own.

### Mediators of Child–Robot Relationship Formation

Several recent CRI studies have dealt with mechanisms that may explain the development of child–robot relationships [e.g., 28, 50]. In line with the S–O–R paradigm, their findings indicate that the emergence of closeness and trust may be mediated by internal states. Two categories of psychological processes can be differentiated, experiential and cognitive ones [e.g., 38]. Accordingly, we differentiate between experiential and cognitive states (for a broader categorization, see e.g., [143]). In the context of CRI, experiential states refer to how children experience, appreciate, and affectively respond to a robot and the interaction with it. Cognitive states refer to children’s perceptions of, and thinking about, a robot during and after their interaction with it. We distinguished cognitive states from experiential rather than from emotional states because the category of experiential states [52] as well as the juxtaposition of experiential and cognitive processes have been established in the literature (see also, in the context of organizational learning, [42]; in the context of psychotherapy, [94]). From a pragmatic perspective, this distinction allowed us to summarize a broader set of psychological responses into one category.

To date, the mediating role of experiential and cognitive states has not been explicitly theorized and investigated in research on the consequences of CRI. However, both experiential and cognitive states are likely to mediate the consequences of children’s interaction with a robot. In each of the aforementioned HRI models, for example, a cognitive state that occurs is social presence [66, 77, 80]. Social presence is a psychological state that applies when “technology users do not notice […] the artificiality of simulated nonhuman social actors” [76, p. 45]. The consistent occurrence of social presence in each of the HRI models points to the relevance of this, and probably also other, cognitive states to HRI in general and to CRI in particular.

Similarly, experiential states can be expected to influence CRI. Generally, affective experiences play a primary role in the development and maintenance of interpersonal relationships (for an overview, see [16], pp. 141–142). Moreover, experiential states are intrinsically rewarding, such that positive subjective experiences induce a desire for repetition [75]. As the development of a relationship by definition requires time, children’s subjective experiences of (repeated) robot encounters are thus likely related to the emergence of child–robot relationships. Against this background, we aim to determine to what extent experiential and cognitive states occur during CRI, how various robot characteristics and interaction styles influence their manifestation, and how experiential and cognitive states relate to closeness and trust.

In sum, we reviewed CRI research on predictors and mediators of child–robot relationship formation in terms of closeness and trust. Specifically, we posed the following research questions: First, we asked which robot characteristics and interaction styles influence closeness and trust. Our second question was whether robot characteristics and interaction styles affect relevant internal states. Third, we asked to what extent internal states elicit closeness and trust. Thus, we aimed to find out whether in the literature there is evidence that internal states may mediate initial influences of robot characteristics and interaction styles on closeness and trust. Because certain concerns regarding the emergence of child–robot relationships are based on robots’ hybrid ontological status [56], our fourth research question asked how children’s interaction with robots differs from their interaction with humans and objects. Fifth, given the importance of biological sex and children’s developmental status for relationship formation [16, 32], we asked to what extent these user characteristics (i.e., children’s age and biological sex) influence closeness, trust, and internal states, as well as the effects of predictors on each of these variables.

### Methodological Characteristics of CRI Research

To date, little is known about the distribution of specific methodological characteristics in CRI research. As a result, the methodological practices and standards in CRI research remain unclear. To judge the internal validity of studies it is necessary to assess their design (i.e., experimental vs. correlational) and general approach (i.e., quantitative vs. qualitative). In correlational research, internal validity is influenced by the time-frame that is investigated. Longitudinal studies generally lend themselves somewhat more to causal interpretations than cross-sectional studies. Finally, the generalizability of the results of the studies depends on their external validity and the composition of their study samples. Therefore, we reported on studies’ (a) design; (b) quantitative or qualitative approach; (c) their cross-sectional or longitudinal character; and (d) sample composition and size.

The aforementioned four characteristics all relate to studies’ more general methodological setup. However, we also know little about specific methodological characteristics of CRI studies, although they may affect both internal and ecological validity (i.e., the generalizability of scientific findings to a societal context). The first two specific methodological characteristics that we will assess refer to the setting of a study in terms of its physical environment (e.g., lab vs. child’s everyday environment) and interaction setting (e.g., individual vs. group interaction). Information about these characteristics is important because both the suitability of the environment and the number of interaction partners affect the likelihood of a child–robot relationship to emerge [40].

A third important specific methodological characteristic is what kind of robot a study uses. As the similarity of both appearance and behavior of interlocutors plays a role in human relationship formation [16], the type of robot likely also influences the formation of child–robot relationships. Similar to Fong [41], we distinguished between anthropomorphic, zoomorphic, caricatured, and functional robots. Fourth and finally, the manner in which a robot is controlled may affect its responsiveness, and thus also the emergence of a child–robot relationship. Many studies rely upon a Wizard of Oz (WOZ) setup. While this setup enables the robot to adequately respond to specific child behaviors [109], it lacks ecological validity. In sum, we thus also reported on studies’ (a) location; (b) interaction setting; (c) robot morphology; and (d) manner of robot control.

## Method

### Database Search

Because CRI is an interdisciplinary field of research, we consulted multiple databases to identify studies relevant to the review: Web of Science (SSCI database), PsycINFO, IEEE Xplore, and ACM DL. In all databases, we searched for both journal articles and conference proceedings. Conference proceedings were included because CRI studies are often published in this format [11, 124]. As limitations in robots’ interactive capacities were overcome only starting in the 2000s [93], we reviewed only studies published between 2000 and 2017. Moreover, to ensure both the comparability within our sample and the generalizability of our findings, we focused only on research with typically-developing (TD) children. Whereas it would theoretically be interesting to compare research with TD children and children with Autism Spectrum Disorder, this was beyond the scope of the present study. Our search and review processes followed the applicable guidelines of the PRISMA (Preferred Reporting Items for Systematic Reviews and Meta-Analyses) statement (i.e., recommendations with respect to the search and selection process and the reporting of eligibility criteria and information sources) [97].

In Web of Science, we searched *topic* (i.e., *title*, *abstract*, *author keywords*, and *keywords plus*) with the search string (child* AND robot* NOT autism NOT *surgery NOT *operative NOT palsy), thus excluding research topics irrelevant to the present study. The same search string was used in IEEE Xplore to search in *metadata only* (i.e., *title*, *abstract*, and *indexing terms*). All publishers were selected except for AGU and BIAI because these two relate to geoscience and aerospace. In PsycINFO, we used the search string (child*.ab,id,ti. AND robot*.ab,id,ti. NOT autism.ab,id,ti. NOT surgery.ab,id,ti. NOT operative.ab,id,ti. NOT palsy.ab,id,ti.), which searches the fields *title*, *abstract*, and *key concepts*. The asterisks before surgery and operative were removed as left truncation is unavailable in PsycINFO. The same applies to the database ACM DL, where we only searched in *abstract* as only one field could be selected simultaneously. Our search syntax was (+child* +AND +robot* −autism −AND −surgery −AND −operative −AND −palsy). Search results were limited to ACM publications. In total, 1865 records were found, which were all English-language publications.

### Screening Process

In a first round of screening, we consulted titles and abstracts to ensure that records were related to CRI. In the case of insufficient information in the abstract, we consulted the corresponding full-text document. We excluded records that did not deal with CRI, or did deal with CRI but studied (a) individuals with physical or mental disabilities; (b) children building, programming, or controlling robots; (c) technological rather than interactive aspects of CRI; (d) CRI from a theoretical perspective; (e) interaction with physically disembodied agents only; (f) children’s observation and perception of robots rather than their interaction with them; and (g) human–human communication via telepresence robots, with participants being aware of their human interaction partner. The exclusion of studies on interactions with only physically disembodied agents (i.e., virtual robots) adds to the comparability of our sample, because the physical presence of robots strongly influences people’s responses [63, 87]. Finally, we excluded review studies, records consisting of uninformative materials, and records that occurred twice within or across databases.

After this first round, we once more screened the remaining records based on title and abstract. We opted for a two-step screening process rather than for a database search with more specific search terms in order to ensure that possibly relevant records were not missed. In the second round, we narrowed our sample to records that dealt with closeness (or similar concepts such as ‘connectedness’ or ‘intimacy’), trust, and relevant internal states. In addition to studies that specifically refer to these concepts, we included studies on attitudes or behaviors related to closeness, such as children’s self-disclosure and empathic behaviors. Likewise, we included studies on trust-related concepts such as reliance and compliance. We included all internal states that occurred in studies that specifically investigated closeness and trust or the just mentioned related concepts.

**Fig. 1:**
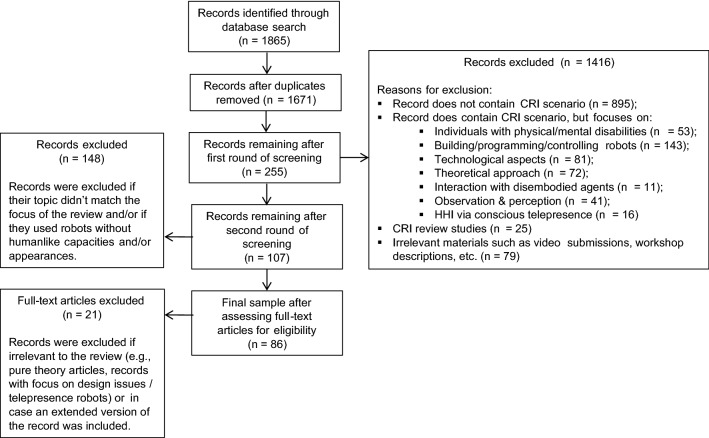
Flow chart of screening process (adapted from PRISMA template)

In the second round of screening, we excluded studies with robots that do not possess humanlike interaction capacities and/or humanlike appearance features. Rather than only including humanoid robots, we thus included all studies using robots that could be argued to have humanlike features. This decision was made to ensure comparability within the sample. That is, it would make little sense to compare relationship formation with humanlike and purely zoomorphic or mechanical robots. The criterion of human likeness is unrelated to the robot characteristic of role (see Sect. 2.1), which more specifically refers to a robot’s role as, for instance, a peer or teacher. Figure 1 shows an information flow chart of the screening process, which was adapted from the template diagram provided in the PRISMA statement [97]. The diagram visualizes the procedure as described above, and specifies numbers of exclusion for each step.

### Interrater Agreement

A second rater decided for 165 records, which were randomly selected from the original sample, whether they should appear in our final sample. The interrater agreement was 98%, and subsequent discussion of the remaining 2% led to agreement upon the first rater’s decisions. As depicted in Fig. 1, the sample was further narrowed down to 86 records that were all published between 2004 and 2017 upon reading the full-text documents.

## Results

### Methodological Characteristics of the Sample

We found 57 quantitative studies and nine qualitative ones. The remaining 20 studies used mixed methods. There were 58 experimental and 28 correlational studies. Sixty studies had a cross-sectional character, 24 studies adopted a longitudinal approach, and two compared single encounters with repeated interactions. Sample sizes ranged from one to 264 participants (*M* = 41.44, *Mdn* = 27, *SD* = 44.28; numbers of participants after exclusion from analyses). All studies relied on convenience samples, which in 48 studies consisted (mainly) of schoolchildren. Of the remaining samples, seven relied on hospital patients and/or children from their social networks; nine on camp, event, or museum visitors; seven on volunteers recruited via flyers, subject pools, bulletin boards, or mailing lists; one on relatives; and one on acquaintances of the researchers. The composition of the remaining 13 samples was unclear. “Appendix 1” provides an overview of the participating children’s age as reported in the studies. In case no age range or mean age was provided, we reported children’s school grade. In most studies (*N *= 67), the age range of the participant sample (partly) fell within the middle childhood age range (i.e., 6–12 years old) [32]. As the reporting of age was heterogeneous, summary statistics could not be calculated.

In terms of location, 41 studies were conducted in a school environment, 14 in a laboratory, and five in hospital settings. The remaining studies took place at various locations (three at museums, two each at camps or play centers, and one each at a university, science fair, chess club, homelike studio environment, or at home). Additionally, three studies used varying settings (switching between home, camp, and/or hospital). Eleven studies did not specify the location. With respect to interactive settings, 69 studies established individual CRI, eight investigated group interactions, and nine relied upon a combination of both. In 64 studies, children interacted with an anthropomorphic robot (see “Appendix 1”). In 40 cases, the anthropomorphic robot used was the Nao robot (or its torso-only version). No studies used functional robots. As for controlling the robot, 53 studies relied either fully or partially on a WOZ setup, whereas in 24 cases a robot operated fully autonomously. Furthermore, three studies compared autonomous behavior and WOZ control. The remaining six were unclear about the robots’ functioning.

In sum, (a) the literature primarily took a quantitative approach; (b) cross-sectional studies (70%) predominated over longitudinal ones; and (c) 67% of the sample adopted an experimental design. (d) About half (48%) of the studies took place in a school environment; (e) 56% of the participant samples consisted of schoolchildren; and (f) in 78% of the studies, participant samples consisted partly or fully of children in middle childhood. Finally, the vast majority of studies (g) investigated dyadic CRI (80%); (h) used an anthropomorphic robot (74%); and (i) relied upon a WOZ setup (62%).

### Effects of Predictors on Closeness and Trust

**Fig. 2:**
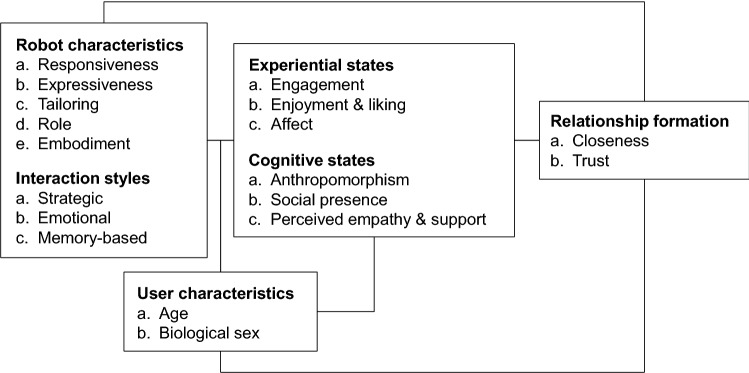
Predictors, mediators, and outcome variables

Our presentation of substantive results will focus on providing an overview of general patterns as well as inconsistencies in the findings of CRI research. It should be noted that, in terms of robot characteristics, tailoring of functionality received minimal attention in the literature reviewed. Therefore, we will include a single predictor ‘tailoring’ that refers to the adaptation of either behavior, appearance, or functionality. Figure 2 provides an overview of the key concepts of this review that we detected in the literature, and how they conceptually relate to our organizational model. “Appendix 2” (see electronic supplementary materials) provides a detailed overview of the concepts assessed in the reviewed studies. In case a conceptual definition was provided, we followed the authors’ interpretation. If not, we relied upon our own interpretation of the available information in each study. We checked, for seven articles, whether a second rater considered them to cover the same concepts as the first rater. Interrater agreement was 85%. Remaining disagreements were resolved through subsequent discussion.

Our first research question was which robot characteristics and interaction styles directly influence closeness and trust. In terms of robot characteristics, a robot’s increased responsiveness tended to stimulate closeness between a child and a robot [4, 88] as well as children’s trust in a robot [23, 49]. Additionally, a robot’s expressiveness generally seemed important to closeness [105, 115, 150], whereas adjusting a robot’s degree of expressiveness did not further increase closeness [65, 70]. Conclusions with respect to influences of tailoring a robot to a child on closeness were inconsistent [1, 28, 99]. In contrast, robots that were presented or perceived in the role of a younger creature with fewer capabilities than the child itself fostered a child’s closeness with the robot [28, 71, 88, 98, 134, 142]. Moreover, closeness equally seemed to be related to the roles assigned to children themselves [31]. Finally, a child’s physical interaction with a robot, rather than embodiment as a feature in itself, seemed to influence closeness [50, 54, 70]. A robot’s embodiment did not influence children’s trust [61, 89].

In general, the effects of robot characteristics on trust were less frequently assessed than the effects of robot characteristics on closeness. Therefore, no valid generalizations can at this point be made regarding the direct influences of robot characteristics on trust. The same holds with respect to the influences of interaction styles on both closeness and trust. Nevertheless, the literature points to a positive association between emotional [4, 88, 136] as well as strategic interaction (albeit less consistently) [4, 5, 20, 21, 57, 59, 60, 73, 81, 127] and closeness between children and robots. Children’s trust in a robot was unaffected by emotional interaction [62, 136] and repeated memory-based interaction [82].

In sum, primarily (a) responsiveness; (b) role; (c) strategic interaction; and (d) emotional interaction elicited feelings of closeness. Responsiveness also seemed to foster trust. Associations between other robot characteristics and interaction styles and trust remained unclear.

### Effects of Predictors on Internal States

Our second research question asked whether robot characteristics and interaction styles would influence children’s internal states. In the studies surveyed, three experiential and three cognitive states could be distinguished (see Fig. 2).

#### Experiential States

Experiential states encompassed (a) engagement; (b) enjoyment and liking; and (c) affect. Engagement can be defined as “active involvement, commitment, and concentrated attention, in contrast to superficial participation, apathy, or lack of interest” [101, p. 11]. Enjoyment and liking refer to children’s positive, pleasant experiences during interaction with a robot. Several studies more specifically addressed children’s emotional responses in terms of affective valence and arousal, which we will therefore discuss separately. While engagement taps into cognitive processes, it is closely related also to enjoyment, appreciation, and affect [148]: Together, these responses constitute one’s general experience of a situation. We, therefore, categorized engagement as an experiential rather than as a cognitive state.

As to the impact of robot characteristics on experiential states, robots’ responsiveness [23, 61, 67, 95, 106, 119, 130, 133, 139] and tailoring [2, 7, 90, 114, 117, 129] primarily seemed to foster children’s engagement. When responsiveness was kept constant, the mode of controlling a robot neither influenced engagement [35] nor enjoyment [140]. While qualitative findings indicate that role was associated with engagement [28, 98, 111], quantitative findings were inconsistent [36, 96, 104]. Embodiment increased observed engagement [61, 89] unless the interaction was more task-oriented [70, 138]. In addition, embodiment positively affected enjoyment and liking [55, 70, 89]. Furthermore, adding a second robot to an interaction seems to positively influence engagement [145] as well as self-reported enjoyment [132].

The effects of expressiveness on experiential states seemed to depend upon the type of expressiveness [62, 68, 115, 128]. In general, robot characteristics had minor or inconsistent influences on children’s affect [69, 104, 106, 115, 118, 128, 147]. With respect to the effects of interaction styles on experiential states, strategic interaction seemed to stimulate engagement [20, 21, 57, 73, 88, 103, 127], as well as enjoyment and liking [20, 47, 60], while emotional and memory-based interaction were positively associated with engagement [2, 4, 29, 53, 82]. Some positive effects of strategic [81, 86] and emotional [21, 78, 136] interaction on affect were also reported. However, a robot’s memory for information that it was not supposed to know caused negative emotional reactions among children [83]. In the long term, combining or expanding interaction styles seemed beneficial to engagement [58, 59, 82].

In sum, all predictors seem to some extent related to (a) engagement, and some were also associated with (b) enjoyment and liking. The effects of predictors on (c) affect were scarce and/or inconsistent.

#### Cognitive States

In the literature reviewed, cognitive states comprised (a) anthropomorphism; (b) social presence; and (c) perceived empathy and support (of the robot toward the child). Anthropomorphism denotes the “attribution of a human form, human characteristics, or human behavior to nonhuman things such as robots […]” [8, p. 74]. Perceived empathy and support were considered cognitive states [as in other categorizations, e.g., 143] as studies generally emphasized their cognitive perception rather than affective experience. As opposed to the more general experiential states that we identified, these cognitive states constitute more specific responses to particular aspects of a situation.

While a robot’s responsiveness tended to foster anthropomorphism ([106] for intelligence; [147] for humanness, but not for intelligence) and perceived support [49, 106], children still considered an unresponsive robot a social agent [10]. Mixed effects of various types of expressiveness on anthropomorphism [6, 70] as well as perceived empathy and support were reported [37, 62]. Effects of tailoring [81, 118] and embodiment [70, 89] on anthropomorphism and social presence were scattered, while a robot’s role was qualitatively related to anthropomorphism [28, 71, 142]. Although strategic and emotional interaction and cognitive states seemed to be associated [3, 57, 59, 81], no clear patterns were detected. However, memory-based interaction seemed to foster anthropomorphism [86, 112]. Unless children’s companionship expectations of a robot were violated [85], social presence remained constant over time across interaction styles [3, 82, 127].

In sum, the effects of predictors on cognitive states were rather inconsistent. However, (a) anthropomorphism as well as (b) perceived empathy and support seemed to be associated with several predictors.

### Effects of Internal States on Closeness and Trust

Our third research question concerned the extent to which cognitive and experiential states elicit closeness and trust. Only one study in the reviewed literature explicitly investigated this impact, reporting a positive correlation between children’s engagement and their motivation to develop a friendly relationship to a robot [59].

In order to see whether the reviewed literature may provide circumstantial evidence of the influence of internal states on closeness and trust, we checked whether a high level of a certain state was consistently accompanied by a high level of closeness or trust. In that case, indirect evidence would exist that the internal state in question and the outcome may be associated. However, for neither experiential nor cognitive states did we find consistent results. For example, evidence emerged that when children were highly engaged, they also tended to feel close to a robot [28, 54, 133]. Accordingly, when children’s engagement in robot interaction did not vary, their closeness or trust in the robot also remained unchanged [82, 112]. However, other studies report variations in engagement but not in closeness [73, 145] or trust [61, 89]. Conversely, yet others found engagement to remain constant while closeness or trust varied [50, 55]. Thus, a consistent association between engagement, and closeness and trust did not seem to exist. Overall, no firm conclusions can currently be drawn about the relationship between internal states and closeness and trust.

In sum, both direct and indirect evidence of associations between internal states and (a) closeness and (b) trust were lacking.

### Differences in Children’s Interaction with Humans, Robots, and Objects

Our fourth research question asked how children’s interactions with robots differ from their interactions with humans and objects. Children’s closeness to robots seems to lie somewhere between their closeness to humans and inanimate objects [123, 133]. In addition, their interpersonal distancing in response to a robot was different than in response to a human [30]. Children trusted humans more than robots in some contexts, whereas in other situations their trust in robots was greater than, or did not differ from, their trust in humans [17, 18, 120, 123]. Findings for engagement were inconsistent [112, 120, 149], but children enjoyed an interaction more when they interacted with a friend than when they interacted with a robot [122, 123]. However, regardless of whether they interacted with a robot or with an adult, children felt equally comfortable and experienced similar levels of enjoyment [17, 149]. Both enjoyment and affect were more positive in the presence of a robot than when a child was playing on its own [121–123]. The social presence of a human was higher than the social presence of his identical robotic counterpart [102], and children more convincingly categorized a robot as ‘human’ when comparing it to a machine than when comparing it to a toy [46].

In sum, (a) children’s trust in humans, robots and objects depended on how trust was operationalized, while (b) closeness and (c) enjoyment were greater in the presence of friends as compared to robots. Playing with robots evoked greater (d) closeness and (e) enjoyment, as well as (f) more positive affect, than solitary play or playing with inanimate objects.

### Influences of User Characteristics

Our fifth and final research question asked to what extent children’s age and biological sex influence closeness, trust, and internal states, as well as the effects of predictors on each of these variables. As to the effects of predictors, three patterns stood out. First, children in middle childhood (i.e., 6–12 years old) seemed to be more critical of, and sensitive to, a robot’s interaction style than children in early childhood (i.e., 2–6 years old [32]) [83, 86, 104]. Second, children’s tendency to anthropomorphize, as well as influences of anthropomorphic robot features on engagement, seemed to decrease with children’s age [57, 104]. Third, tailoring a robot’s sex to that of the child was associated with more positive experiential states among boys [70, 90, 117]. In terms of internal states, three studies consistently found enjoyment to be higher in 8-year-olds than in 12-year-olds [121–123]. Findings with respect to age-related differences in engagement were inconsistent [9, 20, 58, 85, 90, 95, 104, 129, 132, 145]. No generalizations could be made with respect to influences of age and biological sex on closeness and trust.

In sum, no consistent effects of user characteristics on (a) closeness and (b) trust could be disentangled. However, age seemed to be (c) positively related to sensitivity to interaction styles, but negatively related to both (d) anthropomorphic tendencies and effects and (e) experienced enjoyment. (f) Tailoring a robot’s sex to that of the child positively affected boys’ but not girls’ experiential states.

## Discussion

This study reviewed the CRI literature in terms of predictors and underlying processes of relationship formation between children and social robots. Specifically, we aimed to determine, first, which robot characteristics and interaction styles influence closeness and trust; second, whether these same categories of predictors impact children’s internal states during CRI; third, to what extent internal states may influence closeness and trust; fourth, how children’s interaction with robots differs from their interaction with humans and objects; and fifth, to what extent children’s age and gender influence closeness, trust, and internal states, as well as the effects of predictors on each of these variables.

### Influences of Robot Characteristics and Interaction Styles on Closeness and Trust

Robot characteristics, notably responsiveness and role, as well as strategic and emotional interaction styles, were associated with children’s feelings of greater closeness with a robot. Responsiveness was also related to children’s trust in a robot. However, it remained unclear to what extent other robot characteristics and interaction styles are related to trust. The found influences of responsiveness, role, and strategic and emotional interaction on closeness may be related to four specificities of current CRI research.

First, as technological possibilities in CRI are still somewhat limited, our findings may be influenced by the fact that some robot features can currently better be operationalized than others. The relatively distinct influences of these features may thus partly be explained by their relatively easy operationalization. For instance, a robot’s role can explicitly be claimed by the researcher, or even automatically assumed by children, and hence does not require any technical implementation. In contrast, current robots, at least those that can be used in research with children, may still lack the sophistication to emulate differences in facial, vocal, and gestural expressiveness validly—an idea that is supported by the finding that children preferred a robot to be less expressive in case expressiveness hindered intelligibility [62].

Second, particularly in the context of CRI, salient and unambiguous robot features, such as responsiveness and role, may be more effective than non-salient and ambiguous robot features, which run the risk of being differently perceived and interpreted by children. For instance, the impact of tailoring a robot to a child depends on whether the child is able to notice its own similarity to the robot. The immediate intelligibility of robot features for children may also explain why strategic and emotional interaction were more clearly related to closeness than was memory-based interaction. After all, children may interpret a robot’s capacity to recall previous interactions as a sign of technological advancement (for an illustration, see [2]). Conversely, children may associate strategic and emotional interaction more readily with a robot’s social nature.

Third, some predictors are more central in relationship formation than others, which may reflect in our findings. Responsiveness, in particular, constitutes a social skill that plays a pivotal role in the development of social relationships [e.g., 16]. It thus presents a necessary characteristic when studying children’s relationship formation with social robots and it would be surprising not to find an influence of it. Our finding that the absence of expressiveness hinders closeness indirectly supports this observation as expressiveness is also of major importance to relationship formation [e.g., 16].

Fourth, effects of memory-based interaction seem to be age-dependent [83, 86], which may present an alternative explanation for the finding that memory-based interaction was less clearly related to closeness than strategic and emotional interaction. This alternative explanation is supported by the finding that children’s perceptions of robots as mental, social, and moral others differed between age groups [57]. These differences in perception may influence children’s expectations of their interaction with a robot (see [104] for an illustration), as our categorizations and expectations of others are inherently linked [56]. Yet the interpretation of developmental differences demands caution [57], and future research is needed to clarify their nature.

In sum, closeness between children and robots seems primarily triggered by robot characteristics and interaction styles that do not require the implementation of advanced technologies; can be easily perceived and understood by children; and therefore clearly capture the social character of social robots. For children to feel close to a robot, the sociality of robots needs to be tangible and accessible during the interaction with a social robot.

### Influences of Robot Characteristics and Interaction Styles on Internal States

All robot characteristics and all interaction styles seemed to some extent associated with experiential states in terms of children’s engagement and/or enjoyment and liking of robots as well as CRI in general. The fact that CRI can create experiences that are engaging and enjoyable for children confirms the potential of robots to figure as social actors. Reported influences of predictors, particularly robot characteristics, on affect were few and sometimes ambiguous. The inconsistent results may partly be due to current technological limitations in validly operationalizing characteristics and interaction styles that may elicit affective reactions. At the same time, the question of how children react affectively to interaction with robots also needs more systematic research attention than it currently received.

The effects of robot characteristics and interaction styles on cognitive states were less consistent than the effects on experiential states. One explanation may be that cognitive states are more complex and demanding than engagement and enjoyment. Still, several predictors were in some way associated with intricate cognitive states. Notably, anthropomorphism and perceived empathy and support tended to be related to multiple predictors. This result indicates that children’s perception of a robot as a social, humanlike being may influence the formation of child–robot relationships. Overall, the finding that robot characteristics and interaction styles influence engagement and enjoyment more consistently than affect and cognitive states may indicate that, for children to form social relationships to robots, the interaction with a robot should first and foremost be engaging and enjoyable.

### Influences of Internal States on Closeness and Trust

Virtually no research about the impact of internal states during CRI on children’s closeness and trust toward a robot has been conducted to date. If anything, studies occurred in the broader context of HRI rather than CRI. Nearly all studies have focused on robot-related variables because they currently present the core of research on social robots. Moreover, given that social robotics, and notably CRI, is an emerging field of research, studies primarily focus on children’s direct responses to robot-related variables. Consequently, questions on the processes that underlie potential relations between robot-related variables and children’s closeness with, and trust in, robots have not yet received much attention, neither theoretically nor empirically. Our model for organizing the literature may present a first step toward studying relationship formation and its underlying processes in CRI.

### Children’s Interaction with Humans, Robots, and Objects

The literature suggested that children’s differential trust in adults and robots depends on the operationalization of trust. Children felt closer to friends than to robots and enjoyed being with friends more. However, closeness, enjoyment, and affect were still more positive in the presence of a robot than when children were either alone or playing with ‘inanimate objects.’ These findings dovetail with the idea that robots constitute a hybrid ontological category [56], somewhere between the animate and the inanimate. More generally, the results confirm the notion that robots are social entities with whom children are likely to form social relationships.

### Influences of User Characteristics

Children’s sensitivity to a robot’s interaction style seems to increase with age, while anthropomorphic tendencies and effects follow an opposite pattern. Likewise, enjoyment seems to decrease with age, but the influence of age on engagement remains unclear.

As to children’s biological sex, tailoring a robot’s sex to that of the child mainly appealed to boys. Whereas the literature regularly addressed age and biological sex differences in study outcomes, no studies checked for their moderation effects.

### Critical Evaluation of the Field

The lack of consistent patterns arising from our review and the scarcity of firm conclusions that can be drawn partly reflect the still emerging status of the field of CRI research. Although the reviewed literature provided us with many new insights, the formation of child–robot relationships is often studied idiosyncratically, with a lack of established theoretical frameworks, and with incomparable conceptualizations and operationalizations of key concepts. In this context, and in agreement with the issues that Eyssel has raised for research on HRI [39], three methodological and four more substantive shortcomings stand out.

In terms of methodological shortcomings, many studies adopted, first, a correlational design. As a consequence, the internal validity of the studies is limited. The correlational design of the studies is less of a problem for the causal direction between particular robot-related variables and closeness and trust because only one causal direction is possible (i.e., from robot characteristics to closeness and trust). However, with correlational designs we still lack rigorous knowledge of whether it is only the focal robot-related variable that causes changes in closeness and trust, whether unknown confounders produce the effects, or whether the focal variables interact with other unknown variables.

Second, there is a need for moderator-effect studies that clarify how user characteristics such as children’s age and biological sex moderate the effects of predictors on outcome variables. Such studies would be valuable in going beyond the mere description of sample characteristics and corresponding differential outcomes to discover systematic influences of these characteristics. Third, the reporting of methodological and statistical information varies in terms of both scope and detail, which makes it difficult to resolve, or understand, inconsistencies across studies. This shortcoming may partly be due to the fact that findings are often published as conference proceedings, which allow researchers only limited space to present their findings (see [11] for an overview of reporting issues and recommendations in HRI conference papers).

In terms of substantive shortcomings, first, rigorous and valid conceptualizations of closeness, trust, and internal states are often missing. Notably, studies differ in their conceptualizations of engagement and enjoyment, which points to variations in scholars’ understanding of the terminology and impairs the comparability of the studies. Second, closeness, trust, and internal states are frequently measured by observing behaviors whose relationship to the respective concept is not unequivocal. As a result, the interpretation of observational cues varies. For instance, smiling has been treated as an indication of engagement [e.g., 119], enjoyment [e.g., 136], and social presence [e.g., 69]. This variation in the interpretation of behaviors is not surprising given the numerous possible interpretations of a particular behavior depending on the context in which it occurs. However, when contextual information is not provided, it becomes difficult to compare findings and to draw general conclusions.

Third, self-report and observational measures often lead to different outcomes (for an illustration, see [115]), which further adds to the confusion as to whether contradictory findings, both within and between studies, are indeed incompatible. Fourth and finally, many studies report ceiling effects that may reflect a social desirability bias [98, 118]. These ceiling effects are often interpreted as an indication of children’s meaningfully positive evaluation of the robot or their interaction with it, without considering alternative methodological explanations.

### Directions for Future Research

Given the various aforementioned challenges research on CRI has to face, and reflecting earlier recommendations for improving research on HRI [11, 39], several directions for future research are conceivable. First, clear definitions of relevant concepts need to be developed and coherently applied. This will reduce between-study variations in conceptualizations and operationalizations and increase the comparability of the findings, which is an essential prerequisite for cumulative research.

Second, the field would benefit from the development, and regular use, of a set of standardized self-report and observational measures that are validated among children. Such measures would further increase comparability between different studies’ outcomes. In addition, ascertaining both the validity and reliability of measures would contribute to the conclusive power of individual studies.

Third, there is also room for some more general methodological improvements: For example, the conclusive power of many studies could be increased if they varied one, isolated feature at a time, rather than studying multiple features of CRI simultaneously. In correlational designs, more attention should be paid to the inclusion of control variables to reduce the number of alternative interpretations of findings. Moreover, systematic moderator-effect studies would help unravel, for example, the influences of children’s age and biological sex to elucidate systematic influences of such user characteristics. Finally, more elaborate statistical reporting (e.g., inclusion of effect size information in addition to significance levels) could provide additional insight into studies’ findings.

Fourth, more research is needed that attempts to elucidate the psychological mechanisms underlying children’s relationship formation with robots. Even though various influences of robot characteristics and interaction styles on closeness and trust have been reported, it remains largely unknown how these influences come about and whether certain internal states might have amplifying or inhibitory effects. We used the S–O–R paradigm as a heuristic to conceptually organize our findings and provide insights into plausible relationships between potential predictors, mediators, and outcome variables of child–robot relationship formation. Future work should develop this model further and test it statistically.

Fifth and finally, the effects of CRI are often assessed on the basis of cross-sectional studies. Longitudinal studies often limit themselves to only a few interactions between children and robots. Investigating CRI across a larger number of encounters would enhance our knowledge about how relationships between children and robots develop over time, once novelty effects have worn off [84]. To better predict when novelty effects disappear and children’s familiarization with a robot starts, scholars should not only consider the number of robot encounters but also the duration of encounters, the number of people involved in the interaction, and the complexity of a robot’s behavior [84].

To conclude, CRI research has produced important insights into children’s relationship formation with robots. At the same time, the field is still at an early stage, which comes with several challenges, both at a methodological and at a theoretical level. The aforementioned directions for future research could stimulate the field to mature by bringing forth more coherent and robust findings and by bridging gaps in our understanding of the underlying psychological mechanisms.

### Electronic supplementary material

Below is the link to the electronic supplementary material.

Supplementary material 1 (DOCX 50 kb)

## Appendix 1

See Table 1.

**Table 1:** Overview of sample sizes, age information, and robot morphology

	Sample size	Age information		Robot morphology		
		Age range (years)	*M; SD* (years)	Anthropomorphic	Zoomorphic	Caricatured
Abe et al. [1]	31	5–6	5.75; 4.42	LiPRO		
Ahmad et al. [2]	12	10–12		Nao		
Ahmad et al. [3]	23	10–12		Nao		
Ahmad et al. [4]	23	10–12		Nao		
Alves-Oliveira et al. [5]	51		13.67; 0.71	Nao (torso)		
Asselborn et al. [6]	20	5		Nao		
Barco Albo-Canals and Garriga [7]	14	7				iPod-LEGO
Baxter et al. [9]	32	3 and 4	3.46; 0.40	Nao		
Baxter et al. [10]	15		8.45; 0.52	Nao		
Bethel et al. [17]	60	8–12		Nao		
Bethel et al. [18]	14 and 29	4–5	4.5; 0.5	Nao and Zeno		
Blanson Henkemans et al. [20]	27	7–12	11.04; 1.71	Nao		
Blanson Henkemans et al. [21]	5	8–12		Nao		
Breazeal et al. [23]	17	3–5	4.2; 0.79		Dragonbot	
Canamero and Lewis [28]	17	7–12		Nao		
Castellano et al. [29]	5	8			iCat	
Chandra et al. [30]	40	6–8		Nao (torso)		
Chandra et al. [31]	40	6–8		Nao (torso)		
De Haas et al. [35]	14	7–8	7.75; 0.65	Nao		
De Haas et al. [36]	18	3	3.6; 0.29	Nao		
Deshmukh et al. [37]	31	11–14	12.4; –	Nao (torso)		Emys
Guneysu and Arnrich [46]	59		8.4; 2.2	Nao		
Han and Kim [47]	27	Grade 3		Tiro		
Henkel et al. [49]	30	8–12		Nao		
Hieida et al. [50]	37	5–6		LiPRO		
Hyun and Yoon [53]	43	3–4		iRobiQ		
Jeong et al. [54]	4	5–10			Huggable	
Jones et al. [55]	51	11–13		Nao (torso)		
Kahn et al. [57]	90	9, 12, and 15		Robovie		
Kanda et al. [58]	228	6–7 and 11–12		Robovie		
Kanda et al. [59]	37	10–11		Robovie		
Kanda et al. [60]	31	Grade 6		Robovie-R3		
Kennedy et al. [61]	26		7.9; 0.31	Nao		
Kessens et al. [62]	18	8–9	8.5; 0.5		iCat	
Kim et al. [65]	16	10				Mung
Komatsubara et al. [67]	92	Grade 1–6		Robovie		
Kory Westlund et al. [68]	45	4–7	5.2; 0.77			Tega
Kory Westlund et al. [69]	19	3–7	5.04; 1.23			Tega
Kose-Bagci et al. [70]	66	9–10		Kaspar		
Kozima and Nakagawa [71]	27		4.0; –			Keepon
Kruijff-Korbayova et al. [73]	20	11–14		Nao		
Lee et al. [78]	10	3–5		RQ-TITAN		
Leite et al. [81]	40	8–10			iCat	
Leite et al. [82]	16	8–9	8.5; –		iCat	
Leite and Lehman [83]	28	4–10	6.7; 1.82	Jimmy		
Leite et al. [85]	5	5–15			iCat	
Leite et al. [86]	67	4–10	7; 1.77	Piper		
Looije et al. [88]	17	6–10	8.24; 1.25	Nao		
Looije et al. [89]	10		11.1; –	Nao		
Lücking et al. [90]	12	4.3–5.4	4.8; 0.05	Nao		
Michalowski et al. [95]	116	–	–			Keepon
Michalowski et al. [96]	20	5				Keepon
Nalin et al. [98]	3	5–12		Nao		
Neerincx et al. [99]	55	10–14		Nao		
Nishio et al. [102]	2	4 and 10		Geminoid HI-1		
Oh and Kim [103]	33	11		Tiro		
Okita et al. [104]	36 and 9	4–10		Asimo		
Okita et al. [105]	30	5–7		Asimo		
Park et al. [106]	20 and 54	4–8	6.25 and 5.92; 1.33 and 1.4			Tega
Ros et al. [111]	12	7–12	8; 1.91	Nao		
Ros et al. [112]	84	9–11		Nao		
Sadoughi et al. [114]	40	4–10	6.73; 1.72	Sammy		
Saint-Aimé et al. [115]	11	3–5	4.41; 0.6		Emi	
Sandygulova and O’Hare [117]	74	3–9		Nao		
Sandygulova et al. [118]	76	8, 9, 10, 11 and 12		Nao		
Serholt and Barendregt [119]	30	10–13	11.4; 0.86	Nao (torso)		
Serholt et al. [120]	25	11–15	13; 1.4	Nao (torso)		
Shahid et al. [121]	86	8 and 12			iCat	
Shahid et al. [122]	134	8 and 12			iCat	
Shahid et al. [123]	112 and 144	8 and 12			iCat	
Short et al. [127]	26	5–8			Dragonbot	
Silvera-Tawil et al. [128]	264	–	–	Diamandini		
Simmons and Knight [129]	44	5–9	6.8; 1.8			Keepon
Skantze [130]	–	3–15		Furhat		
Tamura et al. [132]	16	3–5	4.3; 0.86	Sota		
Tanaka et al. [133]	5	1.5–2		QRIO		
Tanaka and Ghosh [134]	12	3–5		Nao		
Tielman et al. [136]	17		8.89; 0.81	Nao		
Tozadore et al. [138]	22	7–11	9.36; 1.24	Nao		
Tozadore et al. [139]	30	11–14		Nao		
Tozadore et al. [140]	82	10–12	10.9; 0.53	Nao		
Turkle et al. [142]	1	10		My Real Baby		
Vázquez et al. [145]	74	4–5, 6–8, and 9–10				Chester and Blink
Wigdor et al. [147]	26	9–11	9.32; –	Nao		
Wood et al. [149]	21	7–9		Kaspar		
Yasumatsu et al. [150]	133	2–8	4.67; 1.24	Nao		
