# Supplementary material for: Child–Robot Relationship Formation: A Narrative Review of Empirical Research
Source: Int J Soc Robot. 2019 Jul 3;12(2):325–44. doi: 10.1007/s12369-019-00569-0 (PMC7235061; doi:10.1007/s12369-019-00569-0)
Supplement: Supplementary file 1 — Supplementary material 1 (DOCX 50 kb) [file 12369_2019_569_MOESM1_ESM.docx]

**Table 1.** Overview of Outcomes Measured per Study

|  | Experiential states | | |  | Cognitive states | | |  | Relationship formation | |
| --- | --- | --- | --- | --- | --- | --- | --- | --- | --- | --- |
|  | Engagement | Enjoyment/  liking | Affect |  | Anthropmorphism | Social presence | Perceived empathy/  support |  | Closeness | Trust |
| Abe et al. [1] |  |  |  |  |  |  |  |  | Friendliness, *reported by parents* |  |
| Ahmad et al. [2] | Own interpretation, e.g. “Children also emphasized that the robot should adapt based on previous memory, their emotions, and personality in real-time.” (p. 1), *focus groups* |  |  |  |  |  |  |  |  |  |
| Ahmad et al. [3] |  |  |  |  |  | Social presence, *self-report* | Perceived support, *self-report* |  |  |  |
| Ahmad et al. [4] | Social engagement, *observational* |  |  |  |  |  |  |  | Own interpretation,  “Children felt that the robot was kind and emphatic toward them and it was able to form a bong [sic!] with them in all three conditions” (p. 956), *qualitative observations* |  |
| Alves-Oliveira et al. [5] |  |  |  |  |  |  |  |  | Role assignment (brother/sister, classmate, stranger, relative, friend, parent, tutor, or neighbor), *self-report* |  |
| Asselborn et al. [6] |  |  |  |  | Anthropomorphism, *self-report* |  |  |  |  |  |
| Barco et al. [7] | Engagement, *self-report* |  |  |  |  |  |  |  |  |  |
| Baxter et al. [9] | Engagement, *observational* |  |  |  |  |  |  |  |  |  |
| Baxter et al. [10] |  |  |  |  |  | Perception as social agent, *observational* |  |  |  |  |
| Bethel et al. [17] |  | Own interpretation, items “Was the interviewer nice?” & “Did you like the interviewer?” (“Study Design”, para. 7), *self-report* | Own interpretation, items “Did the interviewer make you feel uncomfortable?” & “Did the interviewer make you upset?” (“Study Design”, para. 7), *self-report* |  |  |  |  |  |  | Own interpretation, item “Did you trust the interviewer?” (“Study Design”, para. 7), *self-report,* disclosure of (peers) being bullied, *observational* |
| Bethel et al. [18] |  |  |  |  |  |  |  |  |  | Own interpretation, *observations of secret sharing* |
| Blanson Henkemans et al. [20] | Engagement, *observational* | Perceived pleasure, *self-report* |  |  |  |  |  |  | Relatedness, *self-report* |  |
| Blanson Henkemans et al. [21] | Own interpretation,  “The children interacting with the personal robot looked longer and more often at the robot […]. Children interacting with the neutral robot only looked at the robot when it  asked them a quiz question.” (p. 179),  *qualitative observations* |  | Own interpretation,  “Children interacting with the personal robot were cheerful when answering a question correctly. The children interacting with the neutral robot did not respond emotionally […].” (p. 179),  *qualitative observations* |  |  |  |  |  | Own interpretation, “Children working with the personal robot were elaborate in their utterances, used the robot’s name and expressed empathy and were encouraging.” (p. 179), *qualitative observations* |  |
| Breazeal et al. [23] | Own interpretation, *observations of gaze behavior* |  |  |  |  |  |  |  |  | Own interpretation, *observations of children seeking & endorsing information* |
| Canamero and Lewis [28] | Own interpretation, e.g. “[…] all the children were fully engaged throughout the interaction until they were asked to leave, and some tried to delay their departure […] or came back to help Robin […].” (p. 534)*, qualitative observations* |  |  |  | Own interpretation, e.g. “[…] asking the robot specific questions about what it was feeling  (‘are you tired?’, ‘are you hungry?’)” p. 535), *qualitative observations* |  |  |  | Own interpretation, e.g. “[…] Robin ‘made lots of friends.’ […] all the children showed concern for Robin, becoming socially, cognitively and emotionally invested in the interaction; this shows that our design [promoted] a feeling of familiarity and friendly social interaction.” (p. 533), “The fact that the children themselves, rather than Robin, were the “knowledgeable grown-ups” […] did seem to pro-mote a positive bond […].” (p. 534), *qualitative observations* |  |
| Castellano et al. [29] | Engagement, *observational* |  |  |  |  |  |  |  |  |  |
| Chandra et al. [30] |  |  |  |  |  |  |  |  | Interpersonal distance, *observational* |  |
| Chandra et al. [31] |  |  |  |  |  |  |  |  | Self-disclosure, *observational* |  |
| De Haas et al. [35] | Immersion, *self-report* |  |  |  |  |  |  |  |  |  |
| De Haas et al. [36] | Engagement, *observational* |  |  |  |  |  |  |  |  |  |
| Deshmukh et al. [37] |  |  |  |  |  |  | Empathy, *self-report* |  |  |  |
| Guneysu and Arnrich [46] |  |  |  |  |  | Machine-toy-human-likeness, *self-report* |  |  |  |  |
| Han and Kim [47] |  | Own interpretation, “The most popular is TIRO’s praising and cheering up service that enhances the relationship between children and TIRO” (p. 256), *qualitative observations* |  |  |  |  |  |  |  |  |
| Henkel et al. [49] |  |  |  |  |  |  | Awareness & understanding: “How well do you think the robot understood how you felt?” (p. 244), *self-report* |  |  | Advice & confidence: “Do you think that the robot could give you advice if you had a problem?” & “Are there any things you could  talk to the robot about that you couldn’t talk to other people  about?” (p. 244),  *self-report* |
| Hieida et al. [50] |  |  |  |  |  |  |  |  | Relationship building, *parent report & observational* |  |
| Hyun and Yoon [53] | Own interpretation, “The children displayed their most active reactions when the robot’s eyes changed into a heart shape and their cheeks turned red.” (p, 678), *qualitative observations* |  |  |  |  |  |  |  |  |  |
| Jeong et al. [54] | Own interpretation,  “[…] children who interacted with the robot appeared to be more physically and mentally motivated to engage with it […]” (p. 104), *qualitative observations* |  |  |  |  |  |  |  | Own interpretation,  “[…] children who interacted with the robot […] conveyed more behavioral evidence that they perceived the intervention as a peer they could socially and emotionally connect with.” (p. 104), *qualitative observations* |  |
| Jones et al. [55] | Engagement, *self-report* | Enjoyment, *self-report* |  |  |  |  |  |  |  | Trust, *self-report* |
| Kahn et al. [57] | Own interpretation, *observations of (participant-initiated) verbal and physical behaviors* |  |  |  | Perception as mental other, *self-report* |  |  |  | Perception as social other, item “Can Robovie be your friend?” (p. 309), *self-report* |  |
| Kanda et al. [58] | Own interpretation, *observations of interaction time* |  |  |  |  |  |  |  |  |  |
| Kanda et al. [59] | Own interpretation, *observations of interaction time,* “We believe that this mechanism [i.e., the number of behaviors of the robot increasing over time] contributed to maintaining long-term interaction […].” (p. 8), *qualitative observation* |  |  |  |  | Own interpretation, “Although Robovie is a robot, I feel it has a humanlike presence”, and “When I interacted with Robovie, I felt as if I had interacted with a human friend” (p. 9), *qualitative observations* |  |  | Friendship motivation, *self-report* |  |
| Kanda et al. [60] |  | Enjoyment, *self-report* |  |  |  |  |  |  | Relationship friend & teacher-/friend-like impression, *self-report* |  |
| Kennedy et al. [61] | Own interpretation, *observations of gaze behavior* |  |  |  |  |  |  |  |  | Own interpretation, *observations of compliance* |
| Kessens et al. [62] |  | Fun, *self-report* |  |  |  |  | Empathy, *self-report* |  |  | Trust, *self-report* |
| Kim et al. [65] |  |  |  |  |  |  |  |  | Empathy toward the robot, *observational* |  |
| Komatsubara et al. [67] | Own interpretation, *observations of number asked questions* |  |  |  |  |  |  |  |  |  |
| Kory Westlund et al. [68] | Engagement, *self-report* |  | Expressivity, *self-report* |  |  |  |  |  |  |  |
| Kory Westlund et al. [69] |  |  | Own interpretation, *observations of smiling & laughter*, shyness, *parent reports* |  |  |  |  |  |  |  |
| Kose-Bagci et al. [70] | Involvement, *self-report* | Enjoyment, *self-report* |  |  | Intelligence, *self-report* |  |  |  | Social attraction, *self-report* |  |
| Kozima and Nakagawa [71] |  |  |  |  | Own interpretation, “They often spoke to Keepon as if they believed that it had a ‘mind’. They interpreted Keepon’s responses […] as having communicative meanings within the interpersonal context.” (p.1059), *qualitative observations* |  |  |  | Own interpretation, “[…] one of the major motivations of such spontaneous  actions would stem from what a child had ever done by his or her caregivers i.e., longing for their caregivers.  Keepon's small appearance and fairly restricted capability would facilitate caretaking behavior in the children. Such  behavior would them be copied and/or exchanged in somewhat competitive relationships with others.” (p. 1059),  *qualitative observations* |  |
| Kruijff-Korbayova et al. [73] | Engagement, *self-report*, interest to interact again, *self-report & observational* |  |  |  |  |  |  |  | Relationship perception, *self-report* |  |
| Lee et al. [78] |  |  | Emotional states, *observational* |  |  |  |  |  |  |  |
| Leite et al. [81] |  |  | Experienced emotions, “Children who interacted with the adaptive-empathic version of the robot reported more frequently ‘happiness’ as one of the experienced emotions.” (p. 371), *interview* |  | Undefined, “Most children from the adaptive-empathic condition indicated as the main difference the fact that iCat provides help and comments, or simply that playing with a robot i[s] more fun than playing with their colleagues […]. Conversely, many participants from the neutral and random-empathic conditions mentioned embodiment aspects of the robot as the main difference, and compared the robot’s skills to the skills of a computer […].” (p. 372), *interview* | Undefined, “Also, some participants [from the empathic conditions] mentioned that playing with the iCat is more similar to playing with another person.” (p. 372), *interview* | Undefined, “In both empathic conditions, children often referred to the helping behaviors of the robot as the main difference between playing with the robot when compared to playing with a friend.” (p. 372), *interview* |  |  |  |
| Leite et al. [82] | Engagement, *self-report* |  |  |  |  | Social presence, *self-report* |  |  |  | Help & self-validation, *self-report* |
| Leite and Lehman [83] |  |  | Affect & comfort, *observational* |  |  |  |  |  |  |  |
| Leite et al. [85] | Own interpretation, *qualitative observations of verbal behavior* |  |  |  |  | Social presence, *self-report* |  |  |  |  |
| Leite et al. [86] |  |  | Affect, *observational* |  | Intelligence, *self-report* |  |  |  |  |  |
| Looije et al. [88] | Own interpretation, “Charlie gets many questions about how it works. All children are interested in unpredictable facts about Charlie […] they really wanted to tell the robot about their experiences in between the visits.” (p. 492), *qualitative observations* |  |  |  |  |  |  |  | Relatedness, *self-report,* e.g. “They also see Charlie immediately as a friend, this is shown by having a picture of Charlie above the bed at home (pp2), having lots of empathy for Charlie when it falls (pp9) and more then [sic!] passing interest in how many friends Charlie has (pp11)” (p. 491), “This was the first evaluation the robot received a gift from children, which shows that there is some kind of bond/relationship forming. The […] dependence of the robot on the child when it fell or has to go to another activity [added to this experience].” (p. 495), *qualitative observations* |  |
| Looije et al. [89] | Attention, *observational* | Enjoyment & preference, *self-report* |  |  | Anthropomorphism, “Although the questionnaires showed no difference in anthropomorphisation, behavior like offering help or asking nongame related questions only occurred in sessions with the robot. By giving the robot and the  agent a (different) name and constantly using this to refer to them, children seemed more ready to see them as different  characters and assign them certain character traits.” (p. 722), *self-report &* *qualitative observations* |  |  |  |  | Trust, *self-report* |
| Lücking et al. [90] | Interaction level, *observational* |  |  |  |  |  |  |  |  |  |
| Michalowski et al. [95] | Interactive involvement, observational |  |  |  |  |  |  |  |  |  |
| Michalowski et al. [96] | Engagement, *observational* |  |  |  |  |  |  |  |  |  |
| Nalin et al. [98] | Own interpretation, “However, when interviewed about the age of the robot, most of them claimed that the robot was younger than themselves […] it seems that children, when strongly committed in their role of educating/improving a robotic companion, adapt their behavior to be more aligned with the robot’s behavior […].” (“Discussion and Conclusions”, para. 2-4), *self-report & observational* |  |  |  |  |  |  |  | Own interpretation, “From the questionnaires, it appears that all children felt a strong connection with the robot, they perceived it as a peer, and most of them felt in control of the interaction or sharing control with the robot” (“Discussion and Conclusions”, para. 2), *self-report* |  |
| Neerincx et al. [99] |  |  |  |  |  |  |  |  | Closeness, *observational* |  |
| Nishio et al. [102] |  |  |  |  |  | Personal presence, *observations* |  |  |  |  |
| Oh and Kim [103] | Own interpretation, “To the question ‘What type of robot do  you want to have?’ most children answered ‘a robot that can  communicate like a friend’ […].” (p. 49), *self-report*, *observations of interaction category frequencies* |  |  |  |  |  |  |  |  |  |
| Okita et al. [104] | Engagement, *(qualitative) observations* |  | Affective behavior, *qualitative observations* |  |  |  |  |  |  |  |
| Okita et al. [105] |  |  |  |  |  |  |  |  | Own interpretation, *observations of proxemics* |  |
| Park et al. [106] | Engagement, *observational* |  | Affect, *observational* |  | Perceived intelligence, *self-report* |  | Perceived emotional support, *self-report* |  |  |  |
| Ros et al. [111] | Quality of interaction (i.e., engagement, interest, involvement, attention), *qualitative observations* |  |  |  |  |  |  |  |  |  |
| Ros et al. [112] | Engagement, *observational* |  |  |  | Own interpretation, robot perception as nice, funny, smart, fake, fragile, tender, affectionate, *self-report* |  |  |  | Own interpretation, robot perception as friend, puppy, adult, toy, computer, *self-report* |  |
| Sadoughi et al. [114] | Engagement, *observational* |  |  |  |  |  |  |  |  |  |
| Saint-Aimé et al. [115] | Own interpretation, item “Do you want to see Emi again?” (“Table 1”), *self-report, observations of gaze* | Own interpretation, items “Is Emi kind?” & “Do you like robots?” (“Table 1”), *self-report* | Own interpretation, item “Are you happy to see Emi?” (“Table 1”), *self-report, observations of facial expressions* |  |  |  |  |  | Own interpretation, item “Is Emi your friend?” (“Table 1”), *self-report*, *observations of proxemic and haptic behavior* |  |
| Sandygulova and O’Hare [117] | Own interpretation, *observations of interaction time* |  | Own interpretation, “On a few occasions with boys, the robot did provoke negative reaction for being a girl. […] Such strong reactions were not observed with girls.” (p. 405),  *qualitative observations* |  |  |  |  |  |  |  |
| Sandygulova et al. [118] |  |  | Attitude, *observational* |  | Intelligence, *self-report* |  |  |  |  |  |
| Serholt and Barendregt [119] | Social engagement, *observational* |  |  |  |  |  |  |  |  |  |
| Serholt et al. [120] | Social engagement, *self-report & observational* |  |  |  |  |  |  |  |  | Own interpretation, *observations of requesting help* |
| Shahid et al. [121] |  | Fun, *self-report* | Emotional state, *self-report* |  |  |  |  |  |  |  |
| Shahid et al. [122] |  | Fun, *self-report* | Expressiveness, *observational* |  |  |  |  |  |  |  |
| Shahid et al. [123] |  | Fun, *self-report* | Expressiveness, *observational* |  |  |  |  |  | Connectedness, *observational* | Compliance, *observational* |
| Short et al. [127] | Engagement, *observational* |  |  |  |  | Social presence, *self-report* |  |  | Relationship building, *self-report* |  |
| Silvera-Tawil et al. [128] |  |  | Comfort, *observational* |  |  |  |  |  |  |  |
| Simmons and Knight [129] | Engagement, *observational* |  |  |  |  |  |  |  |  |  |
| Skantze [130] | Own interpretation, *observations of interaction participation* |  |  |  |  |  |  |  |  |  |
| Tamura et al. [132] | Own interpretation, *observations of verbal behavior* | Preference, *self-report & parent-report* |  |  |  |  |  |  |  |  |
| Tanaka et al. [133] | Own interpretation, “By session 11, a simple reflex-like contingency was introduced so that QRIO giggled immediately after being touched on the head. This contingency made clear to the children that the robot was responsive to them and served to initiate interaction episodes across the entire study […].” (p. 17955), *qualitative observations* |  |  |  |  |  |  |  | Own interpretation, “The colorful teddy bear had elicited many hugs in previous observations with children this age. Surprisingly, it was ignored throughout the study. When children touched QRIO, they did so in a very careful manner. Robby, on the other hand, was treated like an inanimate object or block […].” (p. 17956), *qualitative observations* |  |
| Tanaka and Ghosh [134] |  |  |  |  |  |  |  |  | Own interpretation, *observations of caretaking behavior* |  |
| Tielman et al. [136] |  |  | Emotions, *self-report*, Own interpretation, *observations of expressive behavior* |  |  |  |  |  | Own interpretation, “[…] we see that children  particularly like the fact that the affective robot showed its  emotions and that it moved more. They also thought this  robot was fun and nice and they felt friendship. […] For the non-affective robot, the strongest  argument for choosing it was that it was easier to understand.” (p. 413), *qualitative observations* | Trust, *self-report* |
| Tozadore et al. [138] | Own interpretation, *observations of gaze behavior* |  |  |  |  |  |  |  |  |  |
| Tozadore et al. [139] | Interaction quality, *observational* |  |  |  |  |  |  |  |  |  |
| Tozadore et al. [140] |  | Enjoyment, *self-report* |  |  |  |  |  |  |  |  |
| Turkle et al. [142] |  |  |  |  | Own interpretation, e.g. “Melanie believes that Aibo and My Real Baby are sentient and have emotions. She thinks that when we brought the robotic dog and doll to her school ‘they were probably confused about who their mommies and daddies were because they were being handled by so many different people’.” (p. 351), *qualitative observations* |  |  |  | Own interpretation, e.g. “She sees her role with the robots as straightforward; it is maternal” (p. 351) & “Yeah. I think we really got to know each other a lot better. Our relationship, it grows bigger.” (p. 352), *qualitative observations* |  |
| Vázquez et al. [145] | Social engagement, *observational* |  |  |  |  |  |  |  | Own interpretation, *observations of proxemic behavior* |  |
| Wigdor et al. [147] |  |  | Arousal/ stress, *physiological* |  | Humanness & intelligence, *self-report* |  |  |  |  |  |
| Wood et al. [149] | Interest, *self-report,* own interpretation, *observations of gaze & verbal behavior* | Fun, *self-report* |  |  |  |  |  |  |  |  |
| Yasumatsu et al. [150] |  |  |  |  |  |  |  |  | Own interpretation *observations of altruistic (helping) behavior* |  |

*Note.* For reasons of space and intelligibility, we were unable to discuss all empirical findings of the studies covered in the review. Accordingly, only the findings addressed in the review are included in the table. Please refer to the original studies for a complete overview of outcome variables, measures, and findings.
